# The crystal structure of 2-(3-nitro­phen­yl)-1*H*-benzimidazole monohydrate revisited

**DOI:** 10.1107/S2056989025011466

**Published:** 2026-01-06

**Authors:** Daimí González Caballero, Mayra P. Hernández Sánchez, Javier Alcides Ellena, Pedro Henrique de Oliveira Santiago, Estael Ochoa Rodríguez, Julio Duque Rodríguez, Armando A. Paneque Quevedo

**Affiliations:** a Institute of Materials Science and Technology, Havana, Cuba; b Institute of Physics of Sao Carlos, Sao Paulo, Brazil; c Faculty of Chemistry of University of Havana Havana Cuba; Universidad de la Repüblica, Uruguay

**Keywords:** 2-(3-nitro­phen­yl)-1*H*-benzimidazole, single-crystal X-ray study, hydrogen bonding, π–π stacking

## Abstract

The crystal structure of 2-(3-nitro­phen­yl)-1*H*-benzimidazole monohydrate, C_13_H_9_N_3_O_2_·H_2_O, has been investigated by single-crystal X-ray diffraction. The benzimidazole derivative had its structure studied previously, but it was described in the *P*1 space group [Sudha *et al.*, (2023 ▸). *J. Mol. Struct.***1286**, 135584]. Herein, we report a reexamination of this compound, which this time crystallized in the triclinic centrosymmetric space group *P*1. The benzene ring and the benzimidazole group are almost coplanar, with N—C—C—C torsion angles of −2.2 (3) and −5.9 (4)° in the two independent mol­ecules in the unit cell. The crystal structure features N—H⋯O and O—H⋯N hydrogen bonds and offset π–π stacking inter­actions.

## Abstract 2

The crystal structure of the title compound has been redetermined in the triclinic centrosymmetric space group *P*1. The benzene ring and the benzimidazole group are almost coplanar, with N—C—C—C torsion of 2.2 (3) and 5.9 (4)° in the two independent mol­ecules in the unit cell. The crystal structure features N—H⋯O and O—H⋯N hydrogen bonds and offset π–π stacking inter­actions.

## 1. Chemical context

Benzimidazoles are heterocyclic aromatic compounds of fused benzene and imidazole rings, which are very important for their applications in biochemistry and materials science. 2-Phenyl­benzimidazoles are known for their promising applications as pharmacophores. The structure of phenyl­benzimidazoles have been studied extensively due to their biological activities such as anti­cancer (Mostafa *et al.*, 2019 ▸; Huynh *et al.*, 2020 ▸), anti­viral (Ibba *et al.*, 2021 ▸; Tonelli *et al.*, 2010 ▸), anthelminthic (Escala *et al.*, 2020 ▸) and anti­oxidant (Matysiak *et al.*, 2019 ▸; Baldisserotto *et al.*, 2020 ▸). The crystal structural analysis of these compounds is essential for understanding their physicochemical and biological properties.
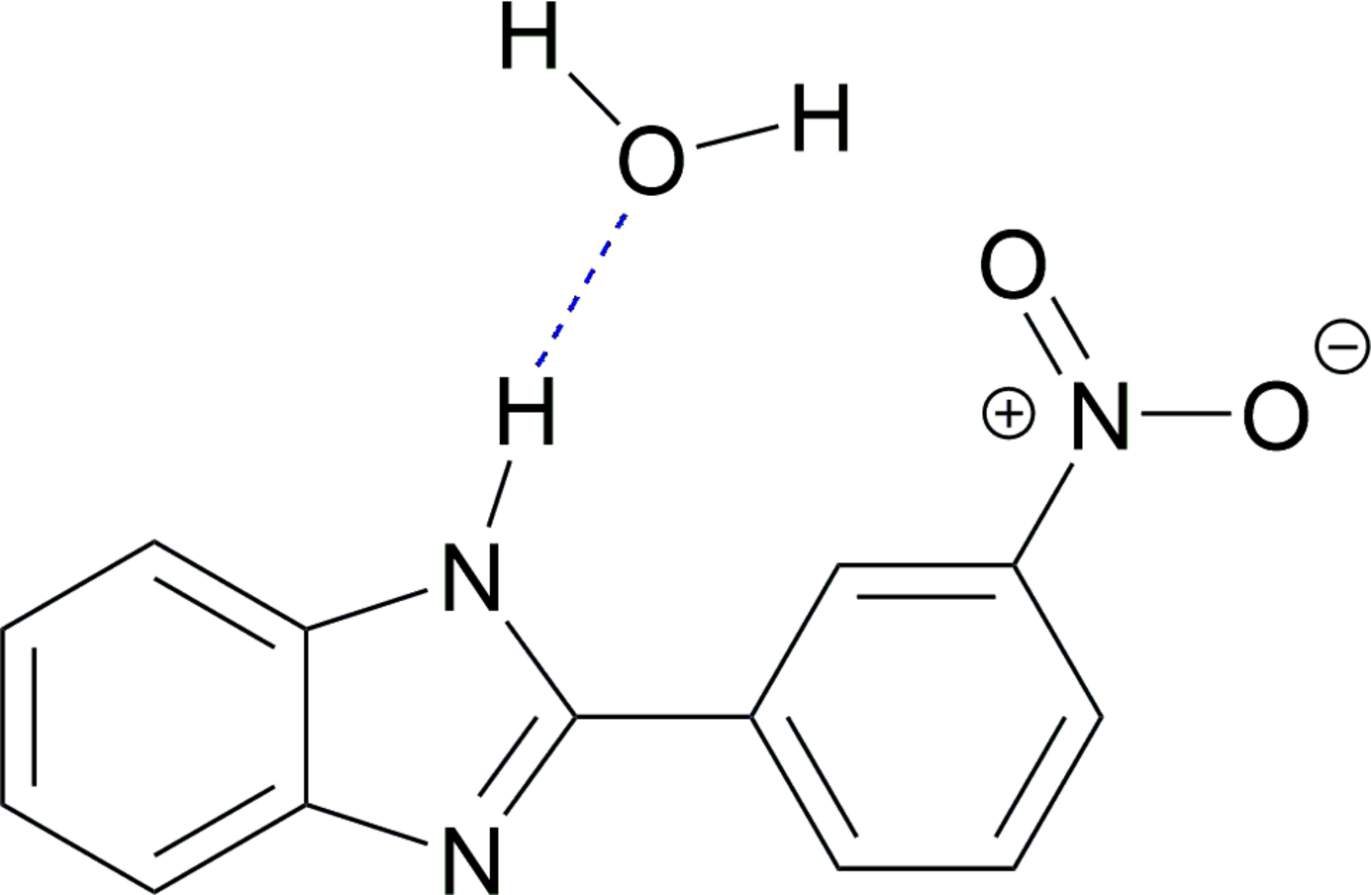


The crystal structure of 2-(3-nitro­phen­yl)-1*H*-benzimidazole was previously reported by Sudha *et al.* (2023 ▸) and deposited in the Cambridge Structural Database [CSD (Groom *et al.*, 2016 ▸) refcode SIBBEO, CCDC No. 2233191] in 2022. The mol­ecule was found to crystallize as a monohydrate in the triclinic crystal system, assigned to the *P*1 space group, with unit-cell parameters: *a* = 7.7366 (2) Å, *b* = 7.7604 (2) Å, *c* = 22.2948 (6) Å, α = 83.2940 (10)°, β = 86.0540 (10)° and γ = 65.7260 (10)° in a volume of 1211.55 (6) Å^3^. The crystal structure featured a network of inter­molecular hydrogen bonds, including C—H⋯O, N—H⋯O, and O—H⋯N inter­actions between the benzimidazole heterocycles and water mol­ecules.

A detailed analysis of the original refinement and the checkCIF *PLATON* report revealed two level G alerts that were unfortunately ignored, specifically, ALERT_2_G ADDSYM Detects New (Pseudo) Centre of Symmetry (97 % Fit) and ALERT_2_G ADDSYM Suggests Possible Pseudo/New Space Group (P-1 Check). These alerts indicated the detection of a new symmetry centre and suggested that the new space group might be *P*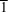. The omission of these alerts motivated us to review the reported structure. Herein, we present a revised crystal structure of 2-(3-nitro­phen­yl)-1*H-*benzimidazole. The compound was synthesized and structurally characterized by single-crystal X-ray diffraction analysis. The refined unit-cell parameters are consistent with those previously reported, confirming that the crystalline phase corresponds to the same compound. However, refinement in the space group *P*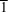 revealed differences in the crystal packing, providing a structural model more consistent with the observed symmetry and tautomerism phenomenon typical of this kind of compound.

## 2. Structural commentary

**Figure 1:**
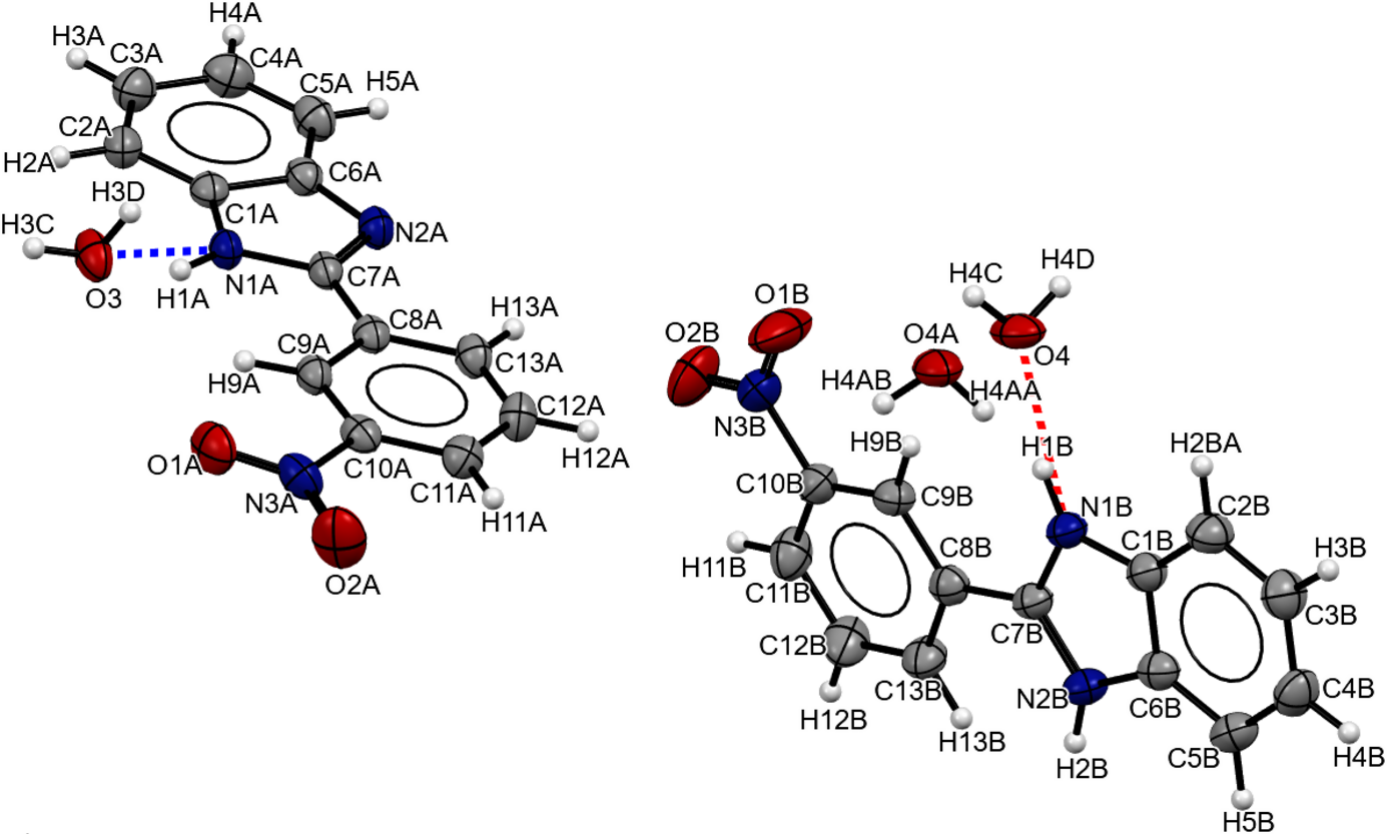
The asymmetric unit of the title compound with displacement ellipsoids drawn at the 50% probability level. Atoms O4 and O4*A* have an occupancy of 0.5.

Single crystals of the title compounds were obtained in reaction of *o*-phenyl­endi­amine and 3-nitro­benzaldehyde. The mol­ecule crystallizes in triclinic space group *P*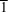 with two mol­ecules of 2-(3-nitro­phen­yl)-1*H*-benzimidazole and two mol­ecules of water in the asymmetric unit, as shown in Fig. 1 ▸. The 2-(3-nitro­phen­yl)-1*H*-benzimidazole mol­ecules are essentially planar, with torsion angles of −2.2 (3)° (N1*A*—C7*A*—C8*A*—C9*A*) and −5.9 (4)° (N1*B*—C7*B*—C8*B*—C9*B*) between the benzene and benzimidazole rings (Table 1 ▸).

One of the benzimidazole mol­ecules has a hydrogen bond to a water mol­ecule and has bond lengths and angles within expected ranges and comparable to those of its nitro-substituted isomers (Li *et al.*, 2005 ▸; Wu *et al.*, 2009 ▸). The other benzimidazole mol­ecule has a hydrogen bond to a disordered water mol­ecule, which has two sets of atomic sites with an occupancy of 0.5. This disorder affects the proton bonded to the nitro­gen atom of the imidazole ring. That is, the bond distances reveal mol­ecules have half a hydrogen atom bonded to the nitro­gen N1*B* and the other half to N2*B*. The C7B—N1B [1.345 (3) Å] and C7B—N2B [1.342 (3) Å] bond lengths are inter­mediate between a single and a double bond (see Table 1 ▸), consistent with tautomerism. This phenomenon has been observed in this family of compounds using fluorescence and UV-vis spectroscopy (Mosquera *et al.*, 1996 ▸).

## 3. Supra­molecular features

**Figure 2:**
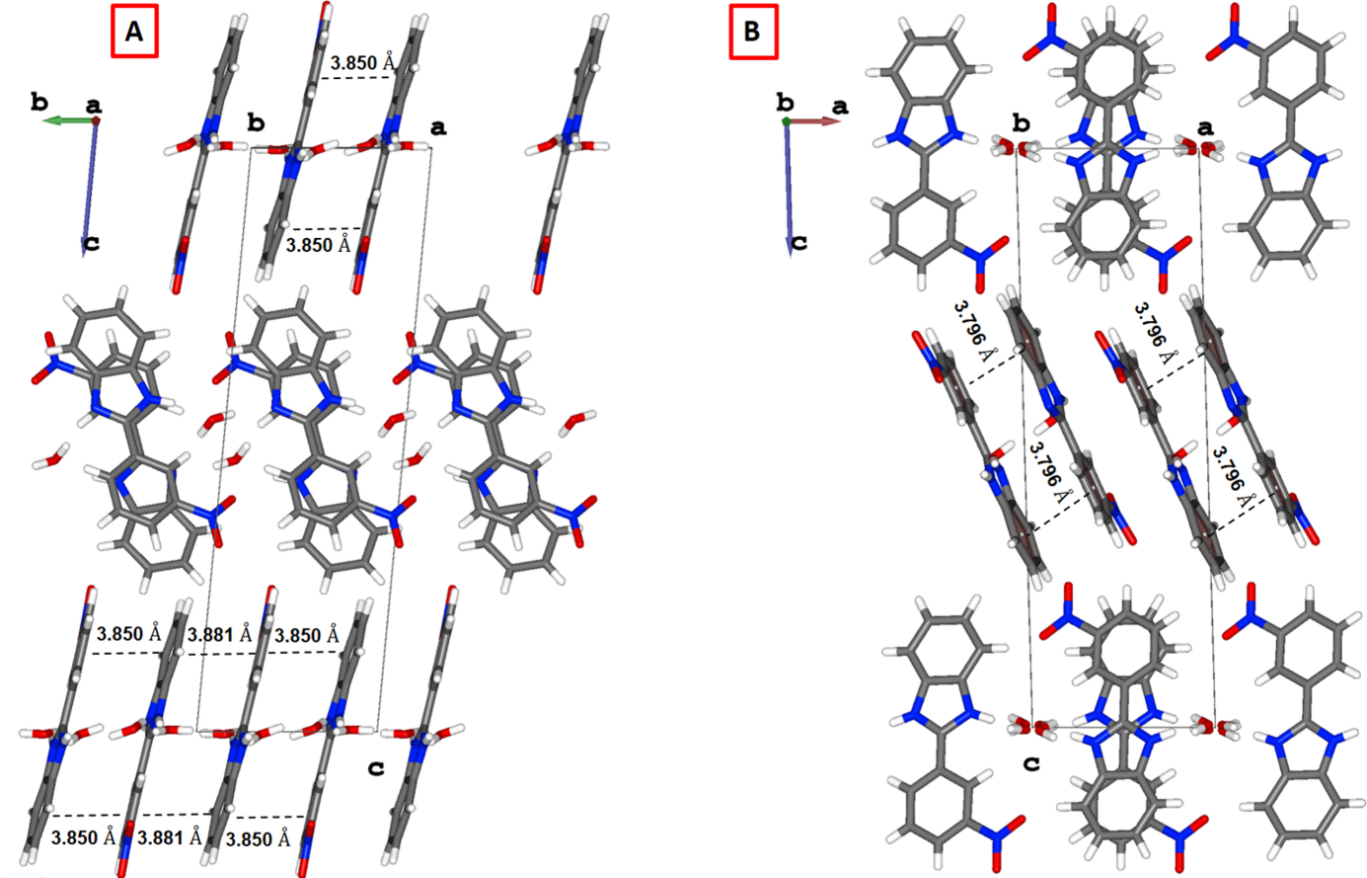
Crystal packing of 2-(3-nitro­phen­yl)-1*H*-benzimidazole. Hydrogen bonds are indicated by dashed lines. (A) Hydrogen-bond inter­actions along the *b* axis and (B) along the *a* axis.

The crystal packing is consolidated by hydrogen-bond inter­actions between the 2-(3-nitro­phen­yl)-1*H*-benzimidazole mol­ecules and the water mol­ecules along two different crystallographic axes. Along the crystallographic *b*-axis, these hydrogen bonds are of the type O3—H3*C*⋯N2*A*, with donor–acceptor bond distances of 2.857 (4) Å (see Fig. 2 ▸, Table 2 ▸). Conversely, along the crystallographic *a*-axis, inter­actions of the type N2*B*—H2*B*⋯O4*A* are observed, with donor–acceptor distances of 2.812 (4) Å (see Fig. 2 ▸, Table 2 ▸).

**Figure 3:**
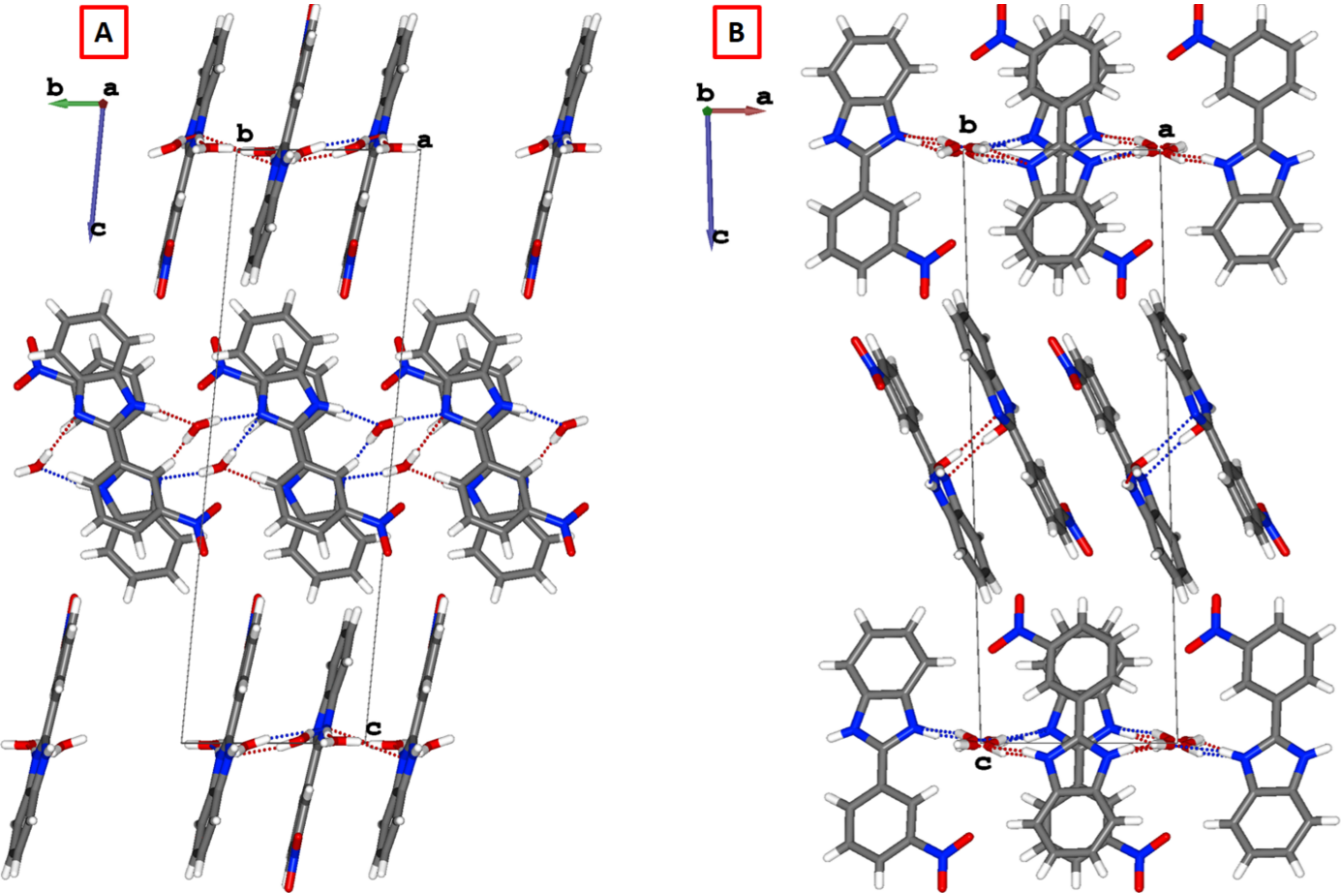
Anti-parallel offset π - π stacking (*A*) along the *b* axis and (*B*) along the *c* axis.

Furthermore, π–π stacking inter­actions are observed between benzene and benzimidazole rings, along to two different directions. Along the *a* axis, the centroid-to-centroid distance is 3.7956 (17) Å, with an inclination angle of 2.96 (9)°, while, along the *b* axis the centroid-to-centroid distances are 3.8810 (19) and 3.8496 (18) Å with inclination angles of 6.99 (10) and 6.88 (10)°, respectively (Fig. 3 ▸).

## 4. Comparative structural analysis

Comparative analysis of both compounds reveals that while the two structures crystallize in the triclinic system and share identical chemical formula and mol­ecular weight, they exhibit differences in their space groups. The lattice parameters show that the crystallographic axes and angles of the previously reported structure present slightly higher values than those determined in this work. These discrepancies can be attributed to differences in measurement conditions, specifically the temperature, as the literature data were recorded at 296 K, whereas our data were obtained at 200 K. This lower temperature induces less vibrational movement of atoms and the cell is determined with better precision. We also observed decreased values of both calculated density and linear absorption coefficient, which could be due to the volume differences of the unit cells. Additional parameters related to model adjustment and refinement show comparable magnitudes in both structural analyses.

The unit cell of the *P*1 structure contains four independent mol­ecules and four water mol­ecules acting as crystallization solvent. These adopt predominantly planar conformations, with torsion angles between the benzimidazole heterocycle and the phenyl ring of 1.2 (6), 3.9 (6), 3.0 (7), and 8.6 (7)°. In contrast, the presence of an inversion center in space group *P*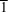 reduces the number of independent mol­ecules to two, with torsion angles of 2.2 (3) and 5.9 (4)°. In this packing arrangement, one water mol­ecule exhibits disorder, suggesting two possible crystalline packings. This disorder facilitated the identification of tautomerism in the benzimidazole mol­ecule, which is clearly reflected in the bond distances of the imidazole ring as discussed above.

The structural analysis of both compounds demonstrates that their solid-state packing is consolidated by a network of inter­molecular hydrogen bonds involving the water mol­ecules and 2-(3-nitro­phen­yl)-1*H*-benzimidazole. In the previously reported structure (space group *P*1), this packing develops predominantly along the crystallographic *a*-axis direction, with donor–acceptor distances of 3.372 (6) Å (C—H⋯O), 2.861 (5) Å (N—H⋯O), and 3.026 (5) Å (O—H⋯N), and corresponding bond angles of 155.7, 157 (4) and 166 (5)°, respectively.

In contrast, the structure proposed in this work (space group *P*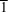) exhibits a more robust packing motif that extends along both the *a*- and *b*- axis directions. This two-dimensional arrangement generates a more extensive hydrogen-bonded network, thereby enhancing the overall packing stability. In this case, the inter­actions are of the types O—H⋯N and N—H⋯O, and the donor–acceptor distances are significantly shorter, not exceeding 2.86 Å, which corresponds to stronger inter­molecular inter­actions.

Additionally, the crystal packing in both compounds features anti­parallel π - π stacking inter­actions between the mol­ecules of 2-(3-nitro­phen­yl)-1*H*-benzimidazole rings. In the previously reported structure, this stacking occurs predominantly along the crystallographic *b* axis, with centroid-to-centroid distances in the range of 3.854–3.914 Å and inclination angles between 1.353 and 1.677°. In parallel, a secondary overlap is observed along the *a* axis, with centroid-to-centroid distances of 3.814–3.819 Å and an inclination angle of 1.449°. Our structure also exhibits this type of π - π stacking inter­action, extending along the *a* and *b*-axis directions. The measured centroid-to-centroid distances of around 3.80 and 3.88 Å are similar to those previously documented.

## 5. Synthesis and crystallization

2-(3-Nitro­phen­yl)-1*H*-benzimidazole was synthesized by refluxing and stirring of *o*-phenyl­endi­amine (10.81 mg, 1 mmol) and 3-nitro­benzaldehyde (15.11 mg, 1 mmol) in 7 mL of aceto­nitrile for 7 h. The reaction progress was monitored for thin layer chromatography (TLC). The resulting solid was separated by vacuum filtration, purified by recrystallization and dried in a desiccator. Single crystals suitable for X-ray diffraction analysis were obtained by slow evaporation from an aceto­nitrile solution over a period of one week. The product was analyzed for NMR.

NMR ^1^H (DMSO *d*_6_, δ ppm): 13.28 (1H; *s*); 9.01 (1H; *m*); 8.61 (1H; *d*; *J* = 7.9 Hz); 8.32 (1H; *dd*; *J* = 8.2; 1.4 Hz); 7.85 (1H; t; *J* = 8.0); 7.5 (2H; *m*); 7.26 (2H; *q*; *J* = 6.0; 3.1 Hz)

NMR ^13^C (DMSO *d*_6_, δ ppm): 149.05; 148.35; 132.47; 131.72; 130.68; 122.65; 124.21; 120.82.

## 6. Refinement

Crystal data, data collection and structure refinement details are summarized in Table 3 ▸. H atoms were located in difference-Fourier maps and refined at idealized positions using a riding model [*U*_iso_(H) = 1.2 or 1.5*U*_eq_].

## Supplementary Material

Crystal structure: contains datablock(s) I. DOI: 10.1107/S2056989025011466/ny2017sup1.cif

Structure factors: contains datablock(s) I. DOI: 10.1107/S2056989025011466/ny2017Isup2.hkl

Supporting information file. DOI: 10.1107/S2056989025011466/ny2017sup3.pdf

Supporting information file. DOI: 10.1107/S2056989025011466/ny2017Isup4.cml

CCDC reference: 2432049

Additional supporting information:  crystallographic information; 3D view; checkCIF report

## Figures and Tables

**Table 1:** Selected geometric parameters (Å, °)

N1*A*—C1*A*	1.377 (3)	N2*A*—C7*A*	1.328 (3)
N1*A*—C7*A*	1.358 (3)	N2*B*—C7*B*	1.342 (3)
N1*B*—C7*B*	1.345 (3)	N2*B*—C6*B*	1.385 (3)
N1*B*—C1*B*	1.386 (3)	O2*A*—N3*A*	1.224 (3)
O1*A*—N3*A*	1.222 (3)	N3*A*—C10*A*	1.470 (3)
N2*A*—C6*A*	1.392 (3)		
			
N1*A*—C7*A*—C8*A*—C9*A*	−2.2 (3)	N1*B*—C7*B*—C8*B*—C9*B*	−5.9 (4)

**Table 2:** Hydrogen-bond geometry (Å, °)

*D*—H⋯*A*	*D*—H	H⋯*A*	*D*⋯*A*	*D*—H⋯*A*
O3—H3*C*⋯N2*A*^i^	0.87	1.99	2.857 (3)	173
N1*A*—H1*A*⋯O3	0.88	2.03	2.849 (3)	154
N1*B*—H1*B*⋯O4	0.88	1.91	2.776 (4)	168
N2*B*—H2*B*⋯O4*A*^ii^	0.88	1.96	2.812 (4)	162

Symmetry codes: (i) 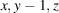; (ii) 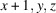.

**Table 3:** Experimental details

Crystal data	
Chemical formula	C_13_H_9_N_3_O_2_·H_2_O
*M*_r_	257.25
Crystal system, space group	Triclinic, *P* 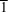
Temperature (K)	200
*a*, *b*, *c* (Å)	7.7169 (1), 7.7279 (1), 22.1425 (4)
α, β, γ (°)	83.171 (2), 85.664 (2), 65.266 (2)
*V* (Å^3^)	1190.34 (4)
*Z*	4
Radiation type	Cu *K*α
μ (mm^−1^)	0.88
Crystal size (mm)	0.26 × 0.16 × 0.08

Data collection	
Diffractometer	XtaLAB Synergy, Dualflex, HyPix
Absorption correction	Multi-scan
*T*_min_, *T*_max_	0.949, 1.000
No. of measured, independent and observed [*I* > 2σ(*I*)] reflections	22169, 5129, 4863
*R*_int_	0.033
(sin θ/λ)_max_ (Å^−1^)	0.638

Refinement	
*R*[*F*^2^ > 2σ(*F*^2^)], *wR*(*F*^2^), *S*	0.060, 0.143, 1.19
No. of reflections	5129
No. of parameters	362
H-atom treatment	H-atom parameters constrained
Δρ_max_, Δρ_min_ (e Å^−3^)	0.26, −0.28

Computer programs: *CrysAlis PRO* (Rigaku OD, 2023 ▸), *SHELXT2018/2* (Sheldrick, 2015*a* ▸), *SHELXL2018/3* (Sheldrick, 2015*b* ▸), *OLEX2* (Dolomanov *et al.*, 2009 ▸) and *Mercury* (Macrae *et al.*, 2020 ▸).

## supplementary crystallographic information

### 2-(3-Nitrophenyl)-1H-benzimidazole monohydrate Crystal data

**Table:**

C_13_H_9_N_3_O_2_·H_2_O	*Z* = 4
*M**_r_* = 257.25	*F*(000) = 536
Triclinic, *P*1	*D*_x_ = 1.435 Mg m^−^^3^
*a* = 7.7169 (1) Å	Cu *K*α radiation, λ = 1.54184 Å
*b* = 7.7279 (1) Å	Cell parameters from 14675 reflections
*c* = 22.1425 (4) Å	θ = 6.0–79.3°
α = 83.171 (2)°	µ = 0.88 mm^−^^1^
β = 85.664 (2)°	*T* = 200 K
γ = 65.266 (2)°	Block, light
*V* = 1190.34 (4) Å^3^	0.26 × 0.16 × 0.08 mm

### 2-(3-Nitrophenyl)-1H-benzimidazole monohydrate Data collection

**Table:**

XtaLAB Synergy, Dualflex, HyPix diffractometer	4863 reflections with *I* > 2σ(*I*)
Detector resolution: 10.0000 pixels mm^-1^	*R*_int_ = 0.033
ω scans	θ_max_ = 79.5°, θ_min_ = 4.0°
Absorption correction: multi-scan	*h* = −6→9
*T*_min_ = 0.949, *T*_max_ = 1.000	*k* = −9→9
22169 measured reflections	*l* = −28→28
5129 independent reflections	

### 2-(3-Nitrophenyl)-1H-benzimidazole monohydrate Refinement

**Table:**

Refinement on *F*^2^	H-atom parameters constrained
Least-squares matrix: full	*w* = 1/[σ^2^(*F*_o_^2^) + (0.0147*P*)^2^ + 1.5554*P*] where *P* = (*F*_o_^2^ + 2*F*_c_^2^)/3
*R*[*F*^2^ > 2σ(*F*^2^)] = 0.060	(Δ/σ)_max_ < 0.001
*wR*(*F*^2^) = 0.143	Δρ_max_ = 0.26 e Å^−^^3^
*S* = 1.19	Δρ_min_ = −0.28 e Å^−^^3^
5129 reflections	Extinction correction: SHELXL-2018/3 (Sheldrick 2015b), Fc^*^=kFc[1+0.001xFc^2^λ^3^/sin(2θ)]^-1/4^
362 parameters	Extinction coefficient: 0.0033 (3)
0 restraints	

### 2-(3-Nitrophenyl)-1H-benzimidazole monohydrate Special details

**Table:**

Geometry. All esds (except the esd in the dihedral angle between two l.s. planes) are estimated using the full covariance matrix. The cell esds are taken into account individually in the estimation of esds in distances, angles and torsion angles; correlations between esds in cell parameters are only used when they are defined by crystal symmetry. An approximate (isotropic) treatment of cell esds is used for estimating esds involving l.s. planes.
Refinement. The data collection was performed at 200 K on a Rigaku Synergy-S Dualflex diffractometer, equipped with an HyPix-6000HE detector and using a CuKα (1.54184 Å) radiation. The CrysAlisPro software was used for the cell refinement, data collection and reduction, and multi-scan absorption correction. The structure was solved by the intrinsic phasing method from SHELXT, and the non-hydrogen atoms were refined considering anisotropic displacement parameters by the full-matrix least-squares on F^2^ method from SHELXL, with both included in Olex2. The hydrogen atoms were located from Fourier difference maps and refined at idealized positions using a riding model [U_iso_(H) = 1.2 or 1.5U_eq_]. Olex2 and Mercury were employed to prepare the material for publication.Data collection: CrysAlis Pro 1.171.43.118a (Rigaku Oxford Diffraction, 2023); cell refinement: CrysAlisPro 1.171.43.118a (Rigaku Oxford Diffraction, 2023); data reduction: CrysAlis Pro 1.171.43.118a (Rigaku Oxford Diffraction, 2023); multi-scan absorption correction: CrysAlisPro (Rigaku Oxford Diffraction, 2023); program(s) used to solve structure: ShelXT 2018/2 (Sheldrick, 2015b); program(s) used to refine structure: ShelXL 2019/2 (Sheldrick, 2015a); interface graphics: Olex2 1.5 (Dolomanov *et al.*, 2009); software used to prepare material for publication: Mercury (Cambridge Crystallographic Data Centre, 2023) and Olex2 1.5 (Dolomanov *et al.*, 2009).The single crystal data for the title compound were submitted to the Cambridge Crystallographic Data Center (CCDC number: 2432049).

### 2-(3-Nitrophenyl)-1H-benzimidazole monohydrate Fractional atomic coordinates and isotropic or equivalent isotropic displacement parameters (Å^2^)

**Table:**

	*x*	*y*	*z*	*U*_iso_*/*U*_eq_	Occ. (<1)
O3	0.1736 (3)	0.0751 (3)	0.46534 (9)	0.0403 (4)	
H3C	0.164738	−0.026839	0.455811	0.060*	
H3D	0.073870	0.128480	0.488832	0.060*	
N1A	0.1889 (3)	0.4351 (3)	0.42963 (9)	0.0283 (4)	
H1A	0.222925	0.310951	0.436817	0.034*	
N1B	0.3379 (3)	0.7820 (3)	1.02554 (9)	0.0304 (4)	
H1B	0.227011	0.792387	1.014005	0.037*	0.5
O1A	0.4550 (3)	−0.0745 (3)	0.60162 (10)	0.0465 (5)	
N2A	0.1397 (3)	0.7325 (3)	0.44496 (9)	0.0301 (4)	
N2B	0.6441 (3)	0.7347 (3)	1.02223 (9)	0.0329 (4)	
H2B	0.761161	0.709550	1.008309	0.039*	0.5
O2A	0.5774 (4)	−0.0252 (3)	0.67853 (10)	0.0596 (6)	
O2B	0.2185 (4)	0.6574 (4)	0.75537 (10)	0.0613 (6)	
N3A	0.4913 (3)	0.0301 (3)	0.63092 (10)	0.0376 (5)	
N3B	0.2103 (3)	0.6730 (3)	0.80954 (11)	0.0412 (5)	
O1B	0.0731 (3)	0.6836 (5)	0.84205 (12)	0.0777 (9)	
C1A	0.1094 (3)	0.5468 (3)	0.37720 (11)	0.0285 (5)	
C6A	0.0788 (3)	0.7335 (3)	0.38728 (11)	0.0287 (5)	
C7A	0.2053 (3)	0.5510 (3)	0.46826 (10)	0.0265 (4)	
C8A	0.2879 (3)	0.4819 (3)	0.52868 (10)	0.0276 (5)	
C10B	0.3731 (4)	0.6794 (4)	0.83806 (12)	0.0339 (5)	
C7B	0.4986 (3)	0.7414 (3)	0.99100 (11)	0.0290 (5)	
C8B	0.5112 (3)	0.7108 (3)	0.92656 (11)	0.0307 (5)	
C9A	0.3495 (3)	0.2896 (3)	0.55094 (11)	0.0293 (5)	
H9A	0.338751	0.199813	0.527338	0.035*	
C10A	0.4261 (3)	0.2330 (3)	0.60782 (11)	0.0295 (5)	
C1B	0.3815 (3)	0.8043 (3)	1.08291 (11)	0.0314 (5)	
C13A	0.3078 (3)	0.6093 (3)	0.56450 (11)	0.0306 (5)	
H13A	0.266785	0.740398	0.549665	0.037*	
C2A	0.0621 (4)	0.5032 (4)	0.32333 (12)	0.0347 (5)	
H2A	0.084461	0.376064	0.316944	0.042*	
C6B	0.5735 (3)	0.7754 (3)	1.08070 (11)	0.0317 (5)	
C11A	0.4476 (4)	0.3572 (4)	0.64416 (11)	0.0336 (5)	
H11A	0.501960	0.313291	0.683143	0.040*	
C9B	0.3581 (3)	0.7054 (3)	0.89880 (11)	0.0317 (5)	
H9B	0.244353	0.719626	0.921598	0.038*	
C5A	−0.0028 (4)	0.8848 (4)	0.34244 (12)	0.0362 (5)	
H5A	−0.024451	1.011989	0.348411	0.043*	
C12A	0.3870 (4)	0.5463 (4)	0.62149 (11)	0.0346 (5)	
H12A	0.399535	0.634844	0.645215	0.042*	
C13B	0.6752 (4)	0.6906 (4)	0.89100 (12)	0.0384 (6)	
H13B	0.781149	0.694671	0.909055	0.046*	
C5B	0.6598 (4)	0.7897 (4)	1.13177 (12)	0.0379 (6)	
H5B	0.789997	0.769988	1.130611	0.045*	
C3A	−0.0190 (4)	0.6546 (4)	0.27960 (12)	0.0396 (6)	
H3A	−0.054207	0.631380	0.242171	0.048*	
C2B	0.2696 (4)	0.8484 (4)	1.13590 (12)	0.0396 (6)	
H2BA	0.139295	0.868184	1.137329	0.048*	
C4B	0.5475 (4)	0.8338 (4)	1.18425 (12)	0.0426 (6)	
H4B	0.601705	0.845371	1.219862	0.051*	
C4A	−0.0507 (4)	0.8418 (4)	0.28922 (12)	0.0404 (6)	
H4A	−0.106953	0.942141	0.258042	0.049*	
C11B	0.5328 (4)	0.6592 (4)	0.80230 (12)	0.0430 (6)	
H11B	0.538312	0.641906	0.760319	0.052*	
C3B	0.3561 (4)	0.8619 (4)	1.18614 (12)	0.0432 (6)	
H3B	0.283808	0.891098	1.223132	0.052*	
C12B	0.6848 (4)	0.6649 (5)	0.82983 (13)	0.0476 (7)	
H12B	0.797537	0.651013	0.806462	0.057*	
O4	−0.0338 (5)	0.8428 (6)	1.0039 (2)	0.0461 (9)	0.5
H4C	−0.099853	0.776852	1.000548	0.069*	0.5
H4D	−0.117933	0.960993	1.002400	0.069*	0.5
O4A	0.0352 (5)	0.6490 (6)	1.0048 (2)	0.0435 (9)	0.5
H4AA	0.110804	0.695421	1.016520	0.065*	0.5
H4AB	0.111454	0.538147	0.993016	0.065*	0.5

### 2-(3-Nitrophenyl)-1H-benzimidazole monohydrate Atomic displacement parameters (Å^2^)

**Table:**

	*U*^11^	*U*^22^	*U*^33^	*U*^12^	*U*^13^	*U*^23^
O3	0.0460 (11)	0.0307 (9)	0.0514 (11)	−0.0228 (8)	0.0142 (8)	−0.0142 (8)
N1A	0.0307 (10)	0.0220 (9)	0.0334 (10)	−0.0112 (7)	0.0003 (8)	−0.0069 (7)
N1B	0.0247 (9)	0.0357 (10)	0.0320 (10)	−0.0130 (8)	−0.0022 (8)	−0.0045 (8)
O1A	0.0507 (11)	0.0275 (9)	0.0630 (13)	−0.0183 (8)	0.0018 (9)	−0.0054 (8)
N2A	0.0339 (10)	0.0251 (9)	0.0336 (10)	−0.0140 (8)	0.0023 (8)	−0.0065 (8)
N2B	0.0255 (9)	0.0387 (11)	0.0354 (11)	−0.0145 (8)	−0.0031 (8)	−0.0015 (9)
O2A	0.0797 (16)	0.0382 (11)	0.0486 (13)	−0.0131 (11)	−0.0161 (11)	0.0073 (9)
O2B	0.0766 (16)	0.0832 (17)	0.0396 (12)	−0.0453 (14)	−0.0113 (11)	−0.0113 (11)
N3A	0.0390 (11)	0.0278 (10)	0.0408 (12)	−0.0100 (9)	0.0061 (9)	−0.0029 (9)
N3B	0.0368 (12)	0.0446 (13)	0.0417 (13)	−0.0138 (10)	−0.0077 (10)	−0.0097 (10)
O1B	0.0372 (12)	0.140 (3)	0.0651 (16)	−0.0377 (15)	0.0009 (11)	−0.0412 (17)
C1A	0.0238 (10)	0.0293 (11)	0.0336 (12)	−0.0119 (9)	0.0030 (9)	−0.0067 (9)
C6A	0.0270 (11)	0.0278 (11)	0.0317 (12)	−0.0114 (9)	0.0035 (9)	−0.0063 (9)
C7A	0.0259 (10)	0.0247 (10)	0.0311 (11)	−0.0122 (9)	0.0053 (8)	−0.0083 (8)
C8A	0.0258 (10)	0.0269 (11)	0.0317 (11)	−0.0126 (9)	0.0050 (9)	−0.0063 (9)
C10B	0.0329 (12)	0.0327 (12)	0.0375 (13)	−0.0140 (10)	−0.0051 (10)	−0.0042 (10)
C7B	0.0258 (11)	0.0299 (11)	0.0323 (12)	−0.0125 (9)	−0.0031 (9)	−0.0014 (9)
C8B	0.0307 (11)	0.0283 (11)	0.0322 (12)	−0.0116 (9)	−0.0014 (9)	−0.0019 (9)
C9A	0.0289 (11)	0.0260 (11)	0.0357 (12)	−0.0135 (9)	0.0039 (9)	−0.0083 (9)
C10A	0.0268 (11)	0.0253 (11)	0.0360 (12)	−0.0115 (9)	0.0055 (9)	−0.0033 (9)
C1B	0.0297 (12)	0.0317 (12)	0.0327 (12)	−0.0126 (9)	−0.0025 (9)	−0.0026 (9)
C13A	0.0348 (12)	0.0266 (11)	0.0336 (12)	−0.0158 (9)	0.0040 (9)	−0.0058 (9)
C2A	0.0339 (12)	0.0338 (12)	0.0379 (13)	−0.0137 (10)	−0.0001 (10)	−0.0111 (10)
C6B	0.0284 (11)	0.0313 (12)	0.0350 (12)	−0.0122 (9)	−0.0018 (9)	−0.0014 (9)
C11A	0.0355 (12)	0.0362 (13)	0.0311 (12)	−0.0170 (10)	0.0009 (10)	−0.0038 (10)
C9B	0.0254 (11)	0.0331 (12)	0.0354 (12)	−0.0110 (9)	0.0001 (9)	−0.0044 (9)
C5A	0.0384 (13)	0.0285 (12)	0.0407 (14)	−0.0135 (10)	0.0026 (10)	−0.0027 (10)
C12A	0.0441 (14)	0.0343 (12)	0.0315 (12)	−0.0215 (11)	0.0034 (10)	−0.0086 (10)
C13B	0.0329 (13)	0.0487 (15)	0.0405 (14)	−0.0226 (11)	0.0046 (10)	−0.0109 (11)
C5B	0.0315 (12)	0.0426 (14)	0.0401 (14)	−0.0157 (11)	−0.0078 (10)	−0.0005 (11)
C3A	0.0373 (13)	0.0464 (15)	0.0346 (13)	−0.0152 (11)	−0.0020 (10)	−0.0091 (11)
C2B	0.0308 (12)	0.0480 (15)	0.0392 (14)	−0.0153 (11)	0.0021 (10)	−0.0068 (11)
C4B	0.0482 (16)	0.0470 (15)	0.0342 (13)	−0.0197 (13)	−0.0111 (11)	−0.0032 (11)
C4A	0.0394 (14)	0.0401 (14)	0.0372 (14)	−0.0134 (11)	−0.0021 (11)	0.0031 (11)
C11B	0.0518 (16)	0.0517 (16)	0.0329 (13)	−0.0278 (13)	0.0060 (11)	−0.0121 (12)
C3B	0.0456 (15)	0.0507 (16)	0.0327 (13)	−0.0188 (13)	0.0037 (11)	−0.0087 (11)
C12B	0.0443 (15)	0.0659 (19)	0.0438 (16)	−0.0327 (14)	0.0145 (12)	−0.0192 (14)
O4	0.0229 (17)	0.043 (2)	0.071 (3)	−0.0129 (15)	−0.0067 (17)	−0.001 (2)
O4A	0.0249 (17)	0.045 (2)	0.061 (3)	−0.0140 (16)	−0.0002 (16)	−0.0096 (18)

### 2-(3-Nitrophenyl)-1H-benzimidazole monohydrate Geometric parameters (Å, º)

**Table:**

O3—H3C	0.8695	C1B—C6B	1.401 (3)
O3—H3D	0.8706	C1B—C2B	1.391 (4)
N1A—H1A	0.8800	C13A—H13A	0.9500
N1A—C1A	1.377 (3)	C13A—C12A	1.387 (3)
N1A—C7A	1.358 (3)	C2A—H2A	0.9500
N1B—H1B	0.8800	C2A—C3A	1.381 (4)
N1B—C7B	1.345 (3)	C6B—C5B	1.393 (3)
N1B—C1B	1.386 (3)	C11A—H11A	0.9500
O1A—N3A	1.222 (3)	C11A—C12A	1.376 (4)
N2A—C6A	1.392 (3)	C9B—H9B	0.9500
N2A—C7A	1.328 (3)	C5A—H5A	0.9500
N2B—H2B	0.8800	C5A—C4A	1.377 (4)
N2B—C7B	1.342 (3)	C12A—H12A	0.9500
N2B—C6B	1.385 (3)	C13B—H13B	0.9500
O2A—N3A	1.224 (3)	C13B—C12B	1.384 (4)
O2B—N3B	1.214 (3)	C5B—H5B	0.9500
N3A—C10A	1.470 (3)	C5B—C4B	1.383 (4)
N3B—O1B	1.213 (3)	C3A—H3A	0.9500
N3B—C10B	1.469 (3)	C3A—C4A	1.403 (4)
C1A—C6A	1.404 (3)	C2B—H2BA	0.9500
C1A—C2A	1.390 (3)	C2B—C3B	1.376 (4)
C6A—C5A	1.397 (3)	C4B—H4B	0.9500
C7A—C8A	1.468 (3)	C4B—C3B	1.399 (4)
C8A—C9A	1.395 (3)	C4A—H4A	0.9500
C8A—C13A	1.398 (3)	C11B—H11B	0.9500
C10B—C9B	1.373 (3)	C11B—C12B	1.382 (4)
C10B—C11B	1.375 (4)	C3B—H3B	0.9500
C7B—C8B	1.463 (3)	C12B—H12B	0.9500
C8B—C9B	1.390 (3)	O4—H4C	0.8699
C8B—C13B	1.400 (3)	O4—H4D	0.8700
C9A—H9A	0.9500	O4A—H4AA	0.8695
C9A—C10A	1.376 (3)	O4A—H4AB	0.8700
C10A—C11A	1.390 (3)		
			
H3C—O3—H3D	104.5	C12A—C13A—H13A	119.7
C1A—N1A—H1A	126.1	C1A—C2A—H2A	121.8
C7A—N1A—H1A	126.1	C3A—C2A—C1A	116.5 (2)
C7A—N1A—C1A	107.73 (19)	C3A—C2A—H2A	121.8
C7B—N1B—H1B	126.8	N2B—C6B—C1B	107.5 (2)
C7B—N1B—C1B	106.38 (19)	N2B—C6B—C5B	131.4 (2)
C1B—N1B—H1B	126.8	C5B—C6B—C1B	121.1 (2)
C7A—N2A—C6A	105.17 (19)	C10A—C11A—H11A	121.3
C7B—N2B—H2B	126.8	C12A—C11A—C10A	117.5 (2)
C7B—N2B—C6B	106.5 (2)	C12A—C11A—H11A	121.3
C6B—N2B—H2B	126.8	C10B—C9B—C8B	119.2 (2)
O1A—N3A—O2A	123.4 (2)	C10B—C9B—H9B	120.4
O1A—N3A—C10A	118.0 (2)	C8B—C9B—H9B	120.4
O2A—N3A—C10A	118.6 (2)	C6A—C5A—H5A	121.4
O2B—N3B—C10B	119.3 (2)	C4A—C5A—C6A	117.3 (2)
O1B—N3B—O2B	123.2 (2)	C4A—C5A—H5A	121.4
O1B—N3B—C10B	117.5 (2)	C13A—C12A—H12A	119.5
N1A—C1A—C6A	105.3 (2)	C11A—C12A—C13A	120.9 (2)
N1A—C1A—C2A	132.1 (2)	C11A—C12A—H12A	119.5
C2A—C1A—C6A	122.6 (2)	C8B—C13B—H13B	119.6
N2A—C6A—C1A	109.5 (2)	C12B—C13B—C8B	120.8 (2)
N2A—C6A—C5A	130.3 (2)	C12B—C13B—H13B	119.6
C5A—C6A—C1A	120.2 (2)	C6B—C5B—H5B	121.5
N1A—C7A—C8A	123.5 (2)	C4B—C5B—C6B	117.0 (2)
N2A—C7A—N1A	112.3 (2)	C4B—C5B—H5B	121.5
N2A—C7A—C8A	124.2 (2)	C2A—C3A—H3A	119.3
C9A—C8A—C7A	121.1 (2)	C2A—C3A—C4A	121.5 (2)
C9A—C8A—C13A	119.0 (2)	C4A—C3A—H3A	119.3
C13A—C8A—C7A	119.8 (2)	C1B—C2B—H2BA	121.5
C9B—C10B—N3B	118.5 (2)	C3B—C2B—C1B	117.0 (2)
C9B—C10B—C11B	123.4 (2)	C3B—C2B—H2BA	121.5
C11B—C10B—N3B	118.1 (2)	C5B—C4B—H4B	119.2
N1B—C7B—C8B	123.5 (2)	C5B—C4B—C3B	121.6 (2)
N2B—C7B—N1B	112.3 (2)	C3B—C4B—H4B	119.2
N2B—C7B—C8B	124.3 (2)	C5A—C4A—C3A	122.1 (2)
C9B—C8B—C7B	120.6 (2)	C5A—C4A—H4A	119.0
C9B—C8B—C13B	118.3 (2)	C3A—C4A—H4A	119.0
C13B—C8B—C7B	121.1 (2)	C10B—C11B—H11B	121.3
C8A—C9A—H9A	120.7	C10B—C11B—C12B	117.4 (2)
C10A—C9A—C8A	118.5 (2)	C12B—C11B—H11B	121.3
C10A—C9A—H9A	120.7	C2B—C3B—C4B	121.8 (3)
C9A—C10A—N3A	118.2 (2)	C2B—C3B—H3B	119.1
C9A—C10A—C11A	123.4 (2)	C4B—C3B—H3B	119.1
C11A—C10A—N3A	118.4 (2)	C13B—C12B—H12B	119.6
N1B—C1B—C6B	107.4 (2)	C11B—C12B—C13B	120.9 (3)
N1B—C1B—C2B	131.0 (2)	C11B—C12B—H12B	119.6
C2B—C1B—C6B	121.6 (2)	H4C—O4—H4D	104.5
C8A—C13A—H13A	119.7	H4AA—O4A—H4AB	104.5
C12A—C13A—C8A	120.7 (2)		
			
N1A—C1A—C6A—N2A	0.1 (2)	C7A—N2A—C6A—C1A	0.3 (2)
N1A—C1A—C6A—C5A	−179.5 (2)	C7A—N2A—C6A—C5A	179.9 (2)
N1A—C1A—C2A—C3A	179.3 (2)	C7A—C8A—C9A—C10A	179.8 (2)
N1A—C7A—C8A—C9A	−2.2 (3)	C7A—C8A—C13A—C12A	−179.5 (2)
N1A—C7A—C8A—C13A	177.0 (2)	C8A—C9A—C10A—N3A	−179.8 (2)
N1B—C7B—C8B—C9B	−5.9 (4)	C8A—C9A—C10A—C11A	−0.8 (3)
N1B—C7B—C8B—C13B	172.6 (2)	C8A—C13A—C12A—C11A	0.0 (4)
N1B—C1B—C6B—N2B	−0.4 (3)	C10B—C11B—C12B—C13B	−0.2 (5)
N1B—C1B—C6B—C5B	179.6 (2)	C7B—N1B—C1B—C6B	0.0 (3)
N1B—C1B—C2B—C3B	−179.5 (3)	C7B—N1B—C1B—C2B	179.5 (3)
O1A—N3A—C10A—C9A	−8.1 (3)	C7B—N2B—C6B—C1B	0.6 (3)
O1A—N3A—C10A—C11A	172.9 (2)	C7B—N2B—C6B—C5B	−179.4 (3)
N2A—C6A—C5A—C4A	−179.5 (2)	C7B—C8B—C9B—C10B	179.0 (2)
N2A—C7A—C8A—C9A	178.3 (2)	C7B—C8B—C13B—C12B	−178.9 (3)
N2A—C7A—C8A—C13A	−2.6 (3)	C8B—C13B—C12B—C11B	0.2 (5)
N2B—C7B—C8B—C9B	175.0 (2)	C9A—C8A—C13A—C12A	−0.3 (3)
N2B—C7B—C8B—C13B	−6.5 (4)	C9A—C10A—C11A—C12A	0.5 (4)
N2B—C6B—C5B—C4B	179.8 (3)	C10A—C11A—C12A—C13A	−0.1 (4)
O2A—N3A—C10A—C9A	172.5 (2)	C1B—N1B—C7B—N2B	0.3 (3)
O2A—N3A—C10A—C11A	−6.6 (3)	C1B—N1B—C7B—C8B	−178.8 (2)
O2B—N3B—C10B—C9B	177.2 (3)	C1B—C6B—C5B—C4B	−0.1 (4)
O2B—N3B—C10B—C11B	−2.7 (4)	C1B—C2B—C3B—C4B	0.3 (4)
N3A—C10A—C11A—C12A	179.5 (2)	C13A—C8A—C9A—C10A	0.7 (3)
N3B—C10B—C9B—C8B	179.7 (2)	C2A—C1A—C6A—N2A	179.9 (2)
N3B—C10B—C11B—C12B	−179.8 (3)	C2A—C1A—C6A—C5A	0.3 (4)
O1B—N3B—C10B—C9B	−2.9 (4)	C2A—C3A—C4A—C5A	0.0 (4)
O1B—N3B—C10B—C11B	177.2 (3)	C6B—N2B—C7B—N1B	−0.6 (3)
C1A—N1A—C7A—N2A	0.7 (3)	C6B—N2B—C7B—C8B	178.6 (2)
C1A—N1A—C7A—C8A	−178.8 (2)	C6B—C1B—C2B—C3B	−0.1 (4)
C1A—C6A—C5A—C4A	0.1 (4)	C6B—C5B—C4B—C3B	0.3 (4)
C1A—C2A—C3A—C4A	0.3 (4)	C9B—C10B—C11B—C12B	0.3 (4)
C6A—N2A—C7A—N1A	−0.7 (3)	C9B—C8B—C13B—C12B	−0.4 (4)
C6A—N2A—C7A—C8A	178.9 (2)	C13B—C8B—C9B—C10B	0.5 (4)
C6A—C1A—C2A—C3A	−0.5 (4)	C5B—C4B—C3B—C2B	−0.5 (5)
C6A—C5A—C4A—C3A	−0.3 (4)	C2B—C1B—C6B—N2B	−179.9 (2)
C7A—N1A—C1A—C6A	−0.5 (2)	C2B—C1B—C6B—C5B	0.0 (4)
C7A—N1A—C1A—C2A	179.7 (2)	C11B—C10B—C9B—C8B	−0.4 (4)

### 2-(3-Nitrophenyl)-1H-benzimidazole monohydrate Hydrogen-bond geometry (Å, º)

**Table:**

*D*—H···*A*	*D*—H	H···*A*	*D*···*A*	*D*—H···*A*
O3—H3*C*···N2*A*^i^	0.87	1.99	2.857 (3)	173
N1*A*—H1*A*···O3	0.88	2.03	2.849 (3)	154
N1*B*—H1*B*···O4	0.88	1.91	2.776 (4)	168
N2*B*—H2*B*···O4*A*^ii^	0.88	1.96	2.812 (4)	162

Symmetry codes: (i) *x*, *y*−1, *z*; (ii) *x*+1, *y*, *z*.
