# Supplementary material for: The crystal structure of 2-(3-nitro­phen­yl)-1H-benzimidazole monohydrate revisited
Source: Acta Crystallogr E Crystallogr Commun. 2026 Jan 6;82(Pt 2):121–5. doi: 10.1107/S2056989025011466 (PMC12874255; doi:10.1107/S2056989025011466)
Supplement: Supplementary file 3 [file e-82-00121-sup3.pdf]

1 supporting information

1.1 Synthesis of single crystal of 2-(3-nitrophenyl)-benzimidazole

2-(3-nitrophenyl)-1H-benzimidazole was synthesized by refluxing and stirring of o-phenylendiamine (10.81 mg, 1 mmol) and 3-nitrobenzaldehyde (15.11 mg, 1 mmol) in 7 mL of acetonitrile for 7 h. The reaction progress was monitored for thin layer chromatography (TLC). The resulting solid was separated by vacuum filtration, purified by recrystallization and dried in a desiccator. Single crystals suitable for X-ray diffraction analysis were obtained by slow evaporation from an acetonitrile solution over a period of two week.

The product was analyzed for NMR.

1.2 Crystal structure analysis

Cristal data

Formula $C_{13}H_9N_3O_2.H_2O$	Z = 4
M_r = 257.25	V = 1190.34(4) $Å^3$
Triclinic system	D_x = 1.435 $Mg\,m^{-3}$
Space group: $P\bar{1}$ (No.2)	Radiation type: CuK_α (λ = 1.54184 Å)
a = 7.71690(10) Å	Cell parameters from 14675 reflections
b = 7.72790(10) Å	F(000) = 536
c = 22.1425(4) Å	T = 199.99(10) K
α = 83.171(2)°	θ = 6.0430 ° - 79.2740 °
β = 85.664(2)°	μ = 0.876 mm^{-1}
γ = 65.266(2)°	Size: 0.24 × 0.13 × 0.05 mm

Data collection

XtaLAB Synergy, Dualflex, HyPix	Measured reflections: 22169
Absorption correction: multi-scan	h = -6 → 9
Method measurement: ω scans	k = -9 → 9
Independent reflections: 5129	l = -28 → 28
Reflections with I > 2σ(I): 4863	θ_{max} = 79.479°
T_{min} = 0.94877 , T_{max} = 1.00000	R_{int} = 0.0329

Refinement

Refinement on F^2

Least-squares matrix: full

$S = 1.186$

Final R indexes $[F_o > 2\sigma(F_o)]$: R1 = 0.0600, wR2 = 0.1421

Final R indexes [all data] R1 = 0.0622, wR2 = 0.1432

Restraints number: 0

Reflections number: 5129

Parameters number: 362

H atom parameters constrained

Primary atom site location: dual

Hydrogen site location: mixed

w $=1/(\sigma^2(F_o^2) + (0.0148P)^2 + 1.5547P)$

where $P = (F_o^2 + 2F_o^2)/3$

$(\Delta\sigma)_{max} = 0.000$

$\Delta\rho_{max} = 0.260$ Å^{-3}

$\Delta\rho_{min} = - 0.284$ Å^{-3}

Geometry: All e.s.d.'s (except the e.s.d. in the dihedral angle between two l.s. planes) are estimated using the full covariance matrix. The cell e.s.d.'s are taken into account individually in the estimation of e.s.d.'s in distances, angles and torsion angles; correlations between e.s.d.'s in cell parameters are only used when they are defined by crystal symmetry. An approximate (isotropic) treatment of cell e.s.d.'s is used for estimating e.s.d.'s involving l.s. planes.

Table 1: Fractional atomic coordinates and isotropic or equivalent isotropic displacement parameters ($Å^2$)

**Átomo**	**x**	**y**	**z**	**U_{iso}*/ U_{eq}**
O3	0.1736(3)	0.0751(3)	0.46534(9)	0.0403(4)
H3C	0.164738	-0.026839	0.455811	0.060 *
H3D	0.073870	0.128480	0.488832	0.060 *
N1A	0.1889(3)	0.4351(3)	0.42963(9)	0.0283(4)
H1A	0.222925	0.310951	0.436817	0.034 *
N1B	0.3379(3)	0.7820(3)	1.02554(9)	0.0304(4)
H1B	0.227011	0.792387	1.014005	0.037 *
O1A	0.4550(3)	-0.0745(3)	0.60162(10)	0.0465(5)
N2A	0.1397(3)	0.7325(3)	0.44496(9)	0.0301(4)
N2B	0.6441(3)	0.7347(3)	1.02223(9)	0.0329(4)
H2B	0.761161	0.709550	1.008309	0.039 *
O2A	0.5774(4)	-0.0252(3)	0.67853(10)	0.0596(6)
O2B	0.2185(4)	0.6574(4)	0.75537(10)	0.0613(6)
N3A	0.4913(3)	0.0301(3)	0.63092(10)	0.0376(5)
N3B	0.2103(3)	0.6730(3)	0.80954(11)	0.0412(5)
O1B	0.0731(3)	0.6836(5)	0.84205(12)	0.0777(9)
C1A	0.1094(3)	0.5468(3)	0.37720(11)	0.0285(5)
C6A	0.0788(3)	0.7335(3)	0.38728(11)	0.0287(5)
C7A	0.2053(3)	0.5510(3)	0.46826(10)	0.0265(4)
C8A	0.2879(3)	0.4819(3)	0.52868(10)	0.0276(5)
C10B	0.3731(4)	0.6794(4)	0.83806(12)	0.0339(5)
C7B	0.4986(3)	0.7414(3)	0.99100(11)	0.0290(5)
C8B	0.5112(3)	0.7108(3)	0.92656(11)	0.0307(5)
C9A	0.3495(3)	0.2896(3)	0.55094(11)	0.0293(5)
H9A	0.338751	0.199813	0.527338	0.035 *
C10A	0.4261(3)	0.2330(3)	0.60782(11)	0.0295(5)
C1B	0.3815(3)	0.8043(3)	1.08291(11)	0.0314(5)
C13A	0.3078(3)	0.6093(3)	0.56450(11)	0.0306(5)
H13A	0.266785	0.740398	0.549665	0.037 *
C2A	0.0621(4)	0.5032(4)	0.32333(12)	0.0347(5)
H2A	0.084461	0.376064	0.316944	0.042 *
C6B	0.5735(3)	0.7754(3)	1.08070(11)	0.0317(5)
C11A	0.4476(4)	0.3572(4)	0.64416(11)	0.0336(5)
H11A	0.501960	0.313291	0.683143	0.040 *
C9B	0.3581(3)	0.7054(3)	0.89880(11)	0.0317(5)
H9B	0.244353	0.719626	0.921598	0.038 *
C5A	-0.0028(4)	0.8848(4)	0.34244(12)	0.0362(5)
H5A	-0.024451	1.011989	0.348411	0.043 *
C12A	0.3870(4)	0.5463(4)	0.62149(11)	0.0346(5)
H12A	0.399535	0.634844	0.645215	0.042 *
C13B	0.6752(4)	0.6906(4)	0.89100(12)	0.0384(6)
H13B	0.781149	0.694671	0.909055	0.046 *
C5B	0.6598(4)	0.7897(4)	1.13177(12)	0.0379(6)
H5B	0.789997	0.769988	1.130611	0.045 *
C3A	-0.0190(4)	0.6546(4)	0.27960(12)	0.0396(6)
H3A	-0.054207	0.631380	0.242171	0.048 *
C2B	0.2696(4)	0.8484(4)	1.13590(12)	0.0396(6)
H2BA	0.139295	0.868184	1.137329	0.048 *
C4B	0.5475(4)	0.8338(4)	1.18425(12)	0.0426(6)
H4B	0.601705	0.845371	1.219862	0.051 *
C4A	-0.0507(4)	0.8418(4)	0.28922(12)	0.0404(6)
H4A	-0.106953	0.942141	0.258042	0.049 *
C11B	0.5328(4)	0.6592(4)	0.80230(12)	0.0430(6)
H11B	0.538312	0.641906	0.760319	0.052 *
C3B	0.3561(4)	0.8619(4)	1.18614(12)	0.0432(6)
H3B	0.283808	0.891098	1.223132	0.052 *
C12B	0.6848(4)	0.6649(5)	0.82983(13)	0.0476(7)
H12B	0.797537	0.651013	0.806462	0.057 *
O4	-0.0338(5)	0.8428(6)	1.0039(2)	0.0461(9)
H4C	-0.099853	0.776852	1.000548	0.069 *
H4D	-0.117933	0.960993	1.002400	0.069 *
O4A	0.0352(5)	0.6490(6)	1.0048(2)	0.0435(9)
H4AA	0.110804	0.695421	1.016520	0.065 *
H4AB	0.111454	0.538147	0.993016	0.065 *

Table 2: Atomic displacement parameters(Å^2)

**Atom**	U^{11}	U^{22}	U^{33}	U^{23}	U^{13}	U^{12}
O3	0.0460(11)	0.0307(9)	0.0514(11)	-0.0142(8)	0.0142(8)	-0.0228(8)
N1A	0.0307(10)	0.0220(9)	0.0334(10)	-0.0069(7)	0.0003(8)	-0.0112(7)
N1B	0.0247(9)	0.0357(10)	0.0320(10)	-0.0045(8)	-0.0022(8)	-0.0130(8)
O1A	0.0507(11)	0.0275(9)	0.0630(13)	-0.0054(8)	0.0018(9)	-0.0183(8)
N2A	0.0339(10)	0.0251(9)	0.0336(10)	-0.0065(8)	0.0023(8)	-0.0140(8)
N2B	0.0255(9)	0.0387(11)	0.0354(11)	-0.0015(9)	-0.0031(8)	-0.0145(8)
O2A	0.0797(16)	0.0382(11)	0.0486(13)	0.0073(9)	-0.0161(11)	-0.0131(11)
O2B	0.0766(16)	0.0832(17)	0.0396(12)	-0.0113(11)	-0.0113(11)	-0.0453(14)
N3A	0.0390(11)	0.0278(10)	0.0408(12)	-0.0029(9)	0.0061(9)	-0.0100(9)
N3B	0.0368(12)	0.0446(13)	0.0417(13)	-0.0097(10)	-0.0077(10)	-0.0138(10)
O1B	0.0372(12)	0.140(3)	0.0651(16)	-0.0412(17)	0.0009(11)	-0.0377(15)
C1A	0.0238(10)	0.0293(11)	0.0336(12)	-0.0067(9)	0.0030(9)	-0.0119(9)
C6A	0.0270(11)	0.0278(11)	0.0317(12)	-0.0063(9)	0.0035(9)	-0.0114(9)
C7A	0.0259(10)	0.0247(10)	0.0311(11)	-0.0083(8)	0.0053(8)	-0.0122(9)
C8A	0.0258(10)	0.0269(11)	0.0317(11)	-0.0063(9)	0.0050(9)	-0.0126(9)
C10B	0.0329(12)	0.0327(12)	0.0375(13)	-0.0042(10)	-0.0051(10)	-0.0140(10)
C7B	0.0258(11)	0.0299(11)	0.0323(12)	-0.0014(9)	-0.0031(9)	-0.0125(9)
C8B	0.0307(11)	0.0283(11)	0.0322(12)	-0.0019(9)	-0.0014(9)	-0.0116(9)
C9A	0.0289(11)	0.0260(11)	0.0357(12)	-0.0083(9)	0.0039(9)	-0.0135(9)
C10A	0.0268(11)	0.0253(11)	0.0360(12)	-0.0033(9)	0.0055(9)	-0.0115(9)
C1B	0.0297(12)	0.0317(12)	0.0327(12)	-0.0026(9)	-0.0025(9)	-0.0126(9)
C13A	0.0348(12)	0.0266(11)	0.0336(12)	-0.0058(9)	0.0040(9)	-0.0158(9)
C2A	0.0339(12)	0.0338(12)	0.0379(13)	-0.0111(10)	-0.0001(10)	-0.0137(10)
C6B	0.0284(11)	0.0313(12)	0.0350(12)	-0.0014(9)	-0.0018(9)	-0.0122(9)
C11A	0.0355(12)	0.0362(13)	0.0311(12)	-0.0038(10)	0.0009(10)	-0.0170(10)
C9B	0.0254(11)	0.0331(12)	0.0354(12)	-0.0044(9)	0.0001(9)	-0.0110(9)
C5A	0.0384(13)	0.0285(12)	0.0407(14)	-0.0027(10)	0.0026(10)	-0.0135(10)
C12A	0.0441(14)	0.0343(12)	0.0315(12)	-0.0086(10)	0.0034(10)	-0.0215(11)
C13B	0.0329(13)	0.0487(15)	0.0405(14)	-0.0109(11)	0.0046(10)	-0.0226(11)
C5B	0.0315(12)	0.0426(14)	0.0401(14)	-0.0005(11)	-0.0078(10)	-0.0157(11)
C3A	0.0373(13)	0.0464(15)	0.0346(13)	-0.0091(11)	-0.0020(10)	-0.0152(11)
C2B	0.0308(12)	0.0480(15)	0.0392(14)	-0.0068(11)	0.0021(10)	-0.0153(11)
C4B	0.0482(16)	0.0470(15)	0.0342(13)	-0.0032(11)	-0.0111(11)	-0.0197(13)
C4A	0.0394(14)	0.0401(14)	0.0372(14)	0.0031(11)	-0.0021(11)	-0.0134(11)
C11B	0.0518(16)	0.0517(16)	0.0329(13)	-0.0121(12)	0.0060(11)	-0.0278(13)
C3B	0.0456(15)	0.0507(16)	0.0327(13)	-0.0087(11)	0.0037(11)	-0.0188(13)
C12B	0.0443(15)	0.0659(19)	0.0438(16)	-0.0192(14)	0.0145(12)	-0.0327(14)
O4	0.0229(17)	0.043(2)	0.071(3)	-0.001(2)	-0.0067(17)	-0.0129(15)
O4A	0.0249(17)	0.045(2)	0.061(3)	-0.0096(18)	-0.0002(16)	-0.0140(16)

Table 3: Bond Lengths

**Atom**	**Atom**	**Length(Å)**
O3	H3C	0.8695
O3	H3D	0.8706
N1A	H1A	0.8800
N1A	C1A	1.377(3)
N1A	C7A	1.358(3)
N1B	H1B	0.8800
N1B	C7B	1.345(3)
N1B	C1B	1.386(3)
O1A	N3A	1.222(3)
N2A	C6A	1.392(3)
N2A	C7A	1.328(3)
N2B	H2B	0.8800
N2B	C7B	1.342(3)
N2B	C6B	1.385(3)
O2A	N3A	1.224(3)
O2B	N3B	1.214(3)
N3A	C10A	1.470(3)
N3B	O1B	1.213(3)
N3B	C10B	1.469(3)
C1A	C6A	1.404(3)
C1A	C2A	1.390(3)
C6A	C5A	1.397(3)
C7A	C8A	1.468(3)
C8A	C9A	1.395(3)
C8A	C13A	1.398(3)
C10B	C9B	1.373(3)
C10B	C11B	1.375(4)
C7B	C8B	1.463(3)
C8B	C9B	1.390(3)
C8B	C13B	1.400(3)
C9A	H9A	0.9500
C9A	C10A	1.376(3)
C10A	C11A	1.390(3)
C1B	C6B	1.401(3)
C1B	C2B	1.391(4)
C13A	H13A	0.9500
C13A	C12A	1.387(3)
C2A	H2A	0.9500
C2A	C3A	1.381(4)
C6B	C5B	1.393(3)
C11A	H11A	0.9500
C11A	C12A	1.376(4)
C9B	H9B	0.9500
C5A	H5A	0.9500
C5A	C4A	1.377(4)
C12A	H12A	0.9500
C13B	H13B	0.9500
C13B	C12B	1.384(4)
C5B	H5B	0.9500
C5B	C4B	1.383(4)
C3A	H3A	0.9500
C3A	C4A	1.403(4)
C2B	H2BA	0.9500
C2B	C3B	1.376(4)
C4B	H4B	0.9500
C4B	C3B	1.399(4)
C4A	H4A	0.9500
C11B	H11B	0.9500
C11B	C12B	1.382(4)
C3B	H3B	0.9500
C12B	H12B	0.9500
O4	H4C	0.8699
O4	H4D	0.8700
O4A	H4AA	0.8695
O4A	H4AB	0.8700

Table 4: Bond Angle

Atom	Atom	Atom	Angle(°)
H3C	O3	H3D	104.5
C1A	N1A	H1A	126.1
C7A	N1A	H1A	126.1
C7A	N1A	C1A	107.73(19)
C7B	N1B	H1B	126.8
C7B	N1B	C1B	106.38(19)
C1B	N1B	H1B	126.8
C7A	N2A	C6A	105.17(19)
C7B	N2B	H2B	126.8
C7B	N2B	C6B	106.5(2)
C6B	N2B	H2B	126.8
O1A	N3A	O2A	123.4(2)
O1A	N3A	C10A	118.0(2)
O2A	N3A	C10A	118.6(2)
O2B	N3B	C10B	119.3(2)
O1B	N3B	O2B	123.2(2)
O1B	N3B	C10B	117.5(2)
N1A	C1A	C6A	105.3(2)
N1A	C1A	C2A	132.1(2)
C2A	C1A	C6A	122.6(2)
N2A	C6A	C1A	109.5(2)
N2A	C6A	C5A	130.3(2)
C5A	C6A	C1A	120.2(2)
N1A	C7A	C8A	123.5(2)
N2A	C7A	N1A	112.3(2)
N2A	C7A	C8A	124.2(2)
C9A	C8A	C7A	121.1(2)
C9A	C8A	C13A	119.0(2)
C13A	C8A	C7A	119.8(2)
C9B	C10B	N3B	118.5(2)
C9B	C10B	C11B	123.4(2)
C11B	C10B	N3B	118.1(2)
N1B	C7B	C8B	123.5(2)
N2B	C7B	N1B	112.3(2)
N2B	C7B	C8B	124.3(2)
C9B	C8B	C7B	120.6(2)
C9B	C8B	C13B	118.3(2)
C13B	C8B	C7B	121.1(2)
C8A	C9A	H9A	120.7
C10A	C9A	C8A	118.5(2)
C10A	C9A	H9A	120.7
C9A	C10A	N3A	118.2(2)
C9A	C10A	C11A	123.4(2)
C11A	C10A	N3A	118.4(2)
N1B	C1B	C6B	107.4(2)
N1B	C1B	C2B	131.0(2)
C2B	C1B	C6B	121.6(2)
C8A	C13A	H13A	119.7
C12A	C13A	C8A	120.7(2)
C12A	C13A	H13A	119.7
C1A	C2A	H2A	121.8
C3A	C2A	C1A	116.5(2)
C3A	C2A	H2A	121.8
N2B	C6B	C1B	107.5(2)
N2B	C6B	C5B	131.4(2)
C5B	C6B	C1B	121.1(2)
C10A	C11A	H11A	121.3
C12A	C11A	C10A	117.5(2)
C12A	C11A	H11A	121.3
C10B	C9B	C8B	119.2(2)
C10B	C9B	H9B	120.4
C8B	C9B	H9B	120.4
C6A	C5A	H5A	121.4
C4A	C5A	C6A	117.3(2)
C4A	C5A	H5A	121.4
C13A	C12A	H12A	119.5
C11A	C12A	C13A	120.9(2)
C11A	C12A	H12A	119.5
C8B	C13B	H13B	119.6
C12B	C13B	C8B	120.8(2)
C12B	C13B	H13B	119.6
C6B	C5B	H5B	121.5
C4B	C5B	C6B	117.0(2)
C4B	C5B	H5B	121.5
C2A	C3A	H3A	119.3
C2A	C3A	C4A	121.5(2)
C4A	C3A	H3A	119.3
C1B	C2B	H2BA	121.5
C3B	C2B	C1B	117.0(2)
C3B	C2B	H2BA	121.5
C5B	C4B	H4B	119.2
C5B	C4B	C3B	121.6(2)
C3B	C4B	H4B	119.2
C5A	C4A	C3A	122.1(2)
C5A	C4A	H4A	119.0
C3A	C4A	H4A	119.0
C10B	C11B	H11B	121.3
C10B	C11B	C12B	117.4(2)
C12B	C11B	H11B	121.3
C2B	C3B	C4B	121.8(3)
C2B	C3B	H3B	119.1
C4B	C3B	H3B	119.1
C13B	C12B	H12B	119.6
C11B	C12B	C13B	120.9(3)
C11B	C12B	H12B	119.6
H4C	O4	H4D	104.5
H4AA	O4A	H4AB	104.5

Table 5: Torsion of dihedral angles

Atom	Atom	Atom	Atom	Torsion(°)	Atom	Atom	Atom	Atom	Torsion(°)
N1A	C1A	C6A	N2A	0.1(2)	C7A	N2A	C6A	C1A	0.3(2)
N1A	C1A	C6A	C5A	-179.5(2)	C7A	N2A	C6A	C5A	179.9(2)
N1A	C1A	C2A	C3A	179.3(2)	C7A	C8A	C9A	C10A	179.8(2)
N1A	C7A	C8A	C9A	-2.2(3)	C7A	C8A	C13A	C12A	-179.5(2)
N1A	C7A	C8A	C13A	177.0(2)	C8A	C9A	C10A	N3A	-179.8(2)
N1B	C7B	C8B	C9B	-5.9(4)	C8A	C9A	C10A	C11A	-0.8(3)
N1B	C7B	C8B	C13B	172.6(2)	C8A	C13A	C12A	C11A	0.0(4)
N1B	C1B	C6B	N2B	-0.4(3)	C10B	C11B	C12B	C13B	-0.2(5)
N1B	C1B	C6B	C5B	179.6(2)	C7B	N1B	C1B	C6B	0.0(3)
N1B	C1B	C2B	C3B	-179.5(3)	C7B	N1B	C1B	C2B	179.5(3)
O1A	N3A	C10A	C9A	-8.1(3)	C7B	N2B	C6B	C1B	0.6(3)
O1A	N3A	C10A	C11A	172.9(2)	C7B	N2B	C6B	C5B	-179.4(3)
N2A	C6A	C5A	C4A	-179.5(2)	C7B	C8B	C9B	C10B	179.0(2)
N2A	C7A	C8A	C9A	178.3(2)	C7B	C8B	C13B	C12B	-178.9(3)
N2A	C7A	C8A	C13A	-2.6(3)	C8B	C13B	C12B	C11B	0.2(5)
N2B	C7B	C8B	C9B	175.0(2)	C9A	C8A	C13A	C12A	-0.3(3)
N2B	C7B	C8B	C13B	-6.5(4)	C9A	C10A	C11A	C12A	0.5(4)
N2B	C6B	C5B	C4B	179.8(3)	C10A	C11A	C12A	C13A	-0.1(4)
O2A	N3A	C10A	C9A	172.5(2)	C1B	N1B	C7B	N2B	0.3(3)
O2A	N3A	C10A	C11A	-6.6(3)	C1B	N1B	C7B	C8B	-178.8(2)
O2B	N3B	C10B	C9B	177.2(3)	C1B	C6B	C5B	C4B	-0.1(4)
O2B	N3B	C10B	C11B	-2.7(4)	C1B	C2B	C3B	C4B	0.3(4)
N3A	C10A	C11A	C12A	179.5(2)	C13A	C8A	C9A	C10A	0.7(3)
N3B	C10B	C9B	C8B	179.7(2)	C2A	C1A	C6A	N2A	179.9(2)
N3B	C10B	C11B	C12B	-179.8(3)	C2A	C1A	C6A	C5A	0.3(4)
O1B	N3B	C10B	C9B	-2.9(4)	C2A	C3A	C4A	C5A	0.0(4)
O1B	N3B	C10B	C11B	177.2(3)	C6B	N2B	C7B	N1B	-0.6(3)
C1A	N1A	C7A	N2A	0.7(3)	C6B	N2B	C7B	C8B	178.6(2)
C1A	N1A	C7A	C8A	-178.8(2)	C6B	C1B	C2B	C3B	-0.1(4)
C1A	C6A	C5A	C4A	0.1(4)	C6B	C5B	C4B	C3B	0.3(4)
C1A	C2A	C3A	C4A	0.3(4)	C9B	C10B	C11B	C12B	0.3(4)
C6A	N2A	C7A	N1A	-0.7(3)	C9B	C8B	C13B	C12B	-0.4(4)
C6A	N2A	C7A	C8A	178.9(2)	C13B	C8B	C9B	C10B	0.5(4)
C6A	C1A	C2A	C3A	-0.5(4)	C5B	C4B	C3B	C2B	-0.5(5)
C6A	C5A	C4A	C3A	-0.3(4)	C2B	C1B	C6B	N2B	-179.9(2)
C7A	N1A	C1A	C6A	-0.5(2)	C2B	C1B	C6B	C5B	0.0(4)
C7A	N1A	C1A	C2A	179.7(2)	C11B	C10B	C9B	C8B	-0.4(4)

Table 6: Atomic Occupancy

Atom	Occupancy
H4C	0.5
H4D	0.5

Atom	Occupancy
H4AB	0.5
H4AA	0.5

Atom	Occupancy
H2B	0.5
H1B	0.5

Atom	Occupancy
O4	0.5
O4A	0.5

Table 7: Hydrogen-bond geometry (Å , °)

D-H—A	D-H(Å)	H–A(Å)	D—A(Å)	D-H—A(^{o})
O3-H3C···N2A$^{(i)}$	0.87	1.99	2.857(4)	172.9
N1A -H1A···O3	0.88	2.03	2.849(3)	153.6
N1B-H1B···O4	0.88	1.91	2.776(5)	168.0
N2B-H2B···O4A$^{(ii)}$	0.88	1.96	2.812(4)	161.8
Symmetry code:	(i) x, y-1, z			
	(ii) x+1,y,z			

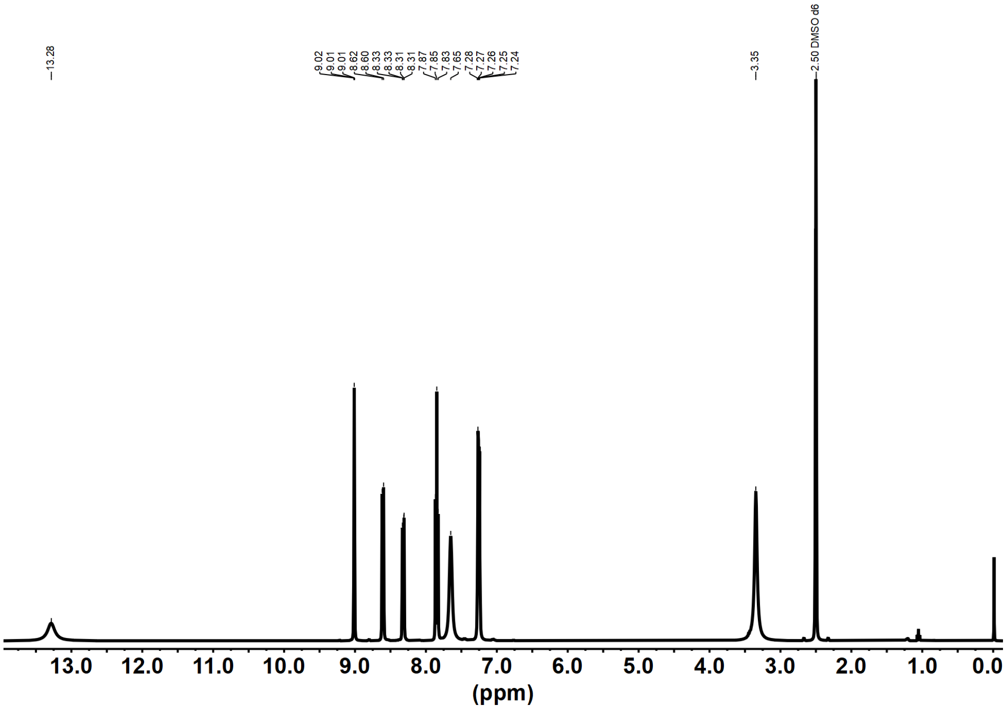

Figure 1: NMR-^{1}H spectra
